# Benzyl­triphenyl­phospho­nium dichlorido­triphenyl­stannate(IV)

**DOI:** 10.1107/S1600536812049689

**Published:** 2012-12-12

**Authors:** Bocar Traore, Mouhamadou Sembene Boye, Mamadou Sidibe, Libasse Diop, Philippe Guionneau

**Affiliations:** a Departement de Chimie, Faculté des Sciences et Techniques, Université Cheikh, Anta Diop, Dakar, Senegal; b CNRS, Université de Bordeaux, ICMCB, UPR 9048, 87 avenue du Dr A. Schweitzer, F-33608 Pessac, France

## Abstract

The crystal structure of the title salt, (C_25_H_22_P)[Sn(C_6_H_5_)_3_Cl_2_] or (PhCH_2_PPh_3_)[SnPh_3_Cl_2_], consists of [PhCH_2_PPh_3_]^+^ cations and [SnPh_3_Cl_2_]^−^ anions in which the Sn^IV^ atom is linked to two Cl atoms and three phenyl groups in a trigonal–bipyramidal geometry, with the Cl atoms in *trans* positions. The cation adopts a tetra­hedral geometry. In the crystal, the cations and the anions are connected by C—H⋯Cl hydrogen bonds, leading to an infinite chain propagating along the *c* direction.

## Related literature

For the [SnPh_3_Cl_2_]^−^ anion, see: Harrison *et al.* (1978 ▶); Ng (1995 ▶). For applications of tin based materials, see: Dutrecq *et al.* (1992 ▶).
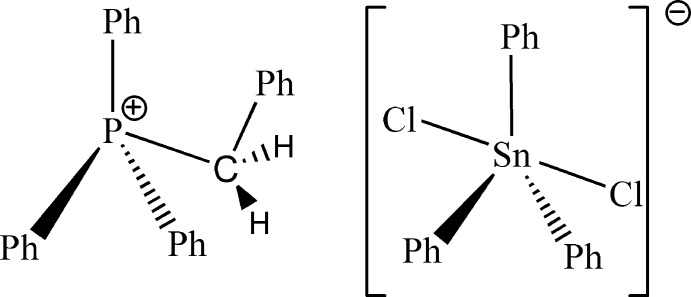


## Experimental

### 

#### Crystal data

(C_25_H_22_P)[Sn(C_6_H_5_)_3_Cl_2_]
*M* *_r_* = 774.29
Monoclinic, 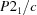

*a* = 10.0222 (2) Å
*b* = 17.1480 (3) Å
*c* = 21.2925 (4) Å
β = 92.042 (1)°
*V* = 3657.02 (12) Å^3^
*Z* = 4
Mo *K*α radiation
μ = 0.92 mm^−1^
*T* = 293 K
0.25 × 0.25 × 0.25 mm

#### Data collection

Nonius KappaCCD diffractometer
Absorption correction: multi-scan (*SCALEPACK*; Otwinowski & Minor, 1997 ▶) *T*_min_ = 0.803, *T*_max_ = 0.803
29025 measured reflections
9370 independent reflections
6243 reflections with *I* > 2σ(*I*)
*R*_int_ = 0.072

#### Refinement

*R*[*F* ^2^ > 2σ(*F* ^2^)] = 0.039
*wR*(*F* ^2^) = 0.102
*S* = 1.03
9370 reflections
425 parameters
H-atom parameters constrained
Δρ_max_ = 0.80 e Å^−3^
Δρ_min_ = −0.76 e Å^−3^

### 

Data collection: *COLLECT* (Nonius, 2003 ▶); cell refinement: *SCALEPACK* (Otwinowski & Minor, 1997 ▶); data reduction: *DENZO* (Otwinowski & Minor, 1997 ▶); program(s) used to solve structure: *SHELXS97* (Sheldrick, 2008 ▶); program(s) used to refine structure: *SHELXL97* (Sheldrick, 2008 ▶); molecular graphics: *ORTEP-3 for Windows* (Farrugia, 2012 ▶); software used to prepare material for publication: *publCIF* (Westrip, 2010 ▶).

## Supplementary Material

Crystal structure: contains datablock(s) I, global. DOI: 10.1107/S1600536812049689/pk2448sup1.cif

Structure factors: contains datablock(s) I. DOI: 10.1107/S1600536812049689/pk2448Isup2.hkl

Additional supplementary materials:  crystallographic information; 3D view; checkCIF report

## Figures and Tables

**Table 1:** Hydrogen-bond geometry (Å, °)

*D*—H⋯*A*	*D*—H	H⋯*A*	*D*⋯*A*	*D*—H⋯*A*
C35—H35⋯Cl2	0.93	2.94	3.696 (3)	139
C37—H37*A*⋯Cl1^i^	0.97	2.84	3.743 (3)	155
C30—H30⋯Cl1^i^	0.93	2.67	3.596 (3)	171

Symmetry code: (i) 
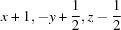
.

**Fig. 1.**
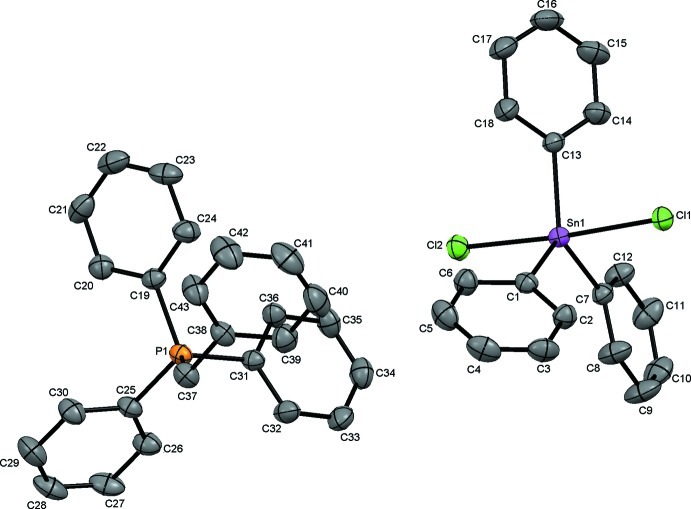
The asymmetric unit of the title compound. Hydrogen atoms have been omitted for clarity. Displacement ellipsoids are drawn at the 30% probability level.

**Fig. 2.**
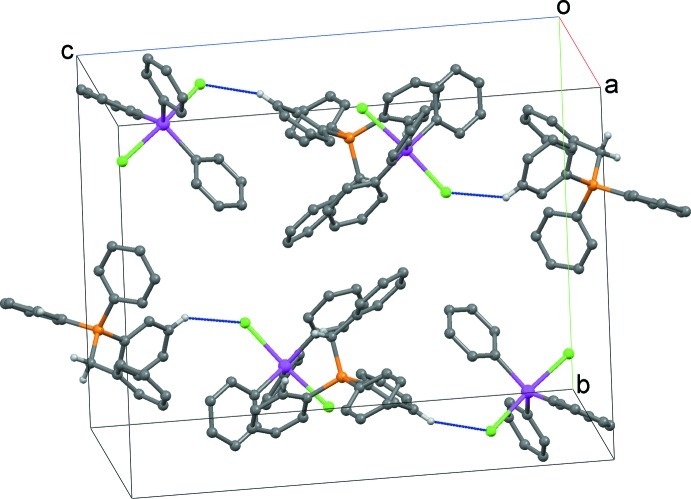
Molecular packing showing hydrogen bonding interactions C—H···Cl (blue lines). H atoms non-participating in hydrogen bonding were omitted for clarity.

## supplementary crystallographic information

### Comment

The structure of [SnPh_3_Cl_2_]^-^ stabilized with different counterions have been reported (Harrison *et al.*, 1978; Ng, 1995). In the scope of our research work toward new organotin (IV) compounds, owing to their various applications: agrochemicals, surface disinfectants, wood preservatives, and marine-antifouling paints (Dutrecq *et al.*, 1992), we report here the crystal structure of [PhCH_2_PPh_3_][SnPh_3_Cl_2_].

In the asymmetric unit of the title compound (Fig. 1), the anion [SnPh_3_Cl_2_]^-^ adopts a trigonal bipyramidal geometry, the chloride atoms occupy the apical positions while the phenyl groups are equatorial. The Sn—C bonds are [2.135 (3); 2.142 (3); 2.153 (3) Å] while the Sn—Cl distances [2.5795 (7); 2.6127 (7) Å] are very similar to the unique Sn—Cl distance value [2.598 (1) Å] (Ng, 1995) but respectively longer and shorter than those [2.573 (7); 2.689 (6) Å] reported (Harrison *et al.*, 1978) within the same anion. The sum of the equatorial angles (360°) is consistent with almost perfectly planar SnPh_3_ residue, while the Cl—Sn—Cl deviates from linearity [175.77 (3)°].

[SnPh_3_Cl_2_]^-^ and [PhCH_2_PPh_3_]^+^ are linked through short C—H···Cl contacts (Table1 & Fig.2).

### Experimental

All chemicals were purchased from Aldrich (Germany) and used without any further purification. The studied adduct is obtained following a two stage reaction. Synthesis of (PhCH_2_PPh_3_)_2_PhAsO_3_: this salt is obtained by neutralization of PhAsO_3_H_2_ (9.899 mmol in water) by PhCH_2_Ph_3_PCl (19.798 mmol in ethanolic solution) according to the following reaction:

PhAsO_3_H_2_ + 2(PhCH_2_PPh_3_Cl) → [(PhCH_2_PPh_3_)_2_PhAsO_3_] + 2HCl

Synthesis of [PhCH_2_PPh_3_][SnPh_3_Cl_2_]: this compound was obtained by mixing ethanolic solutions of (PhCH_2_PPh_3_)_2_PhAsO_3_ (0.5 mmol) and SnPh_3_Cl (1.0 mmoL) in a 1/2 ratio. The mixture was stirred for around two hours at room temperature. Suitable crystals for X-ray diffraction were obtained after slow solvent evaporation. (m.p. 393 K). The title compound was isolated according to the following reaction:

[PhCH_2_PPh_3_]_2_[PhAsO_3_] + 2SnPh_3_Cl → [PhCH_2_PPh_3_][SnPh_3_Cl_2_] + [PhCH_2_PPh_3_.PhAsO_3_][SnPh_3_]

### Refinement

All H atoms were placed in geometrically calculated positions (*d*(C–H)=0.93 Å for phenyl-H and 0.97 Å for methyelene-H) and refined using a riding model with *U*_iso_(H)=1.2*U*_eq_ of the respective carrier atom.

### Crystal data

**Table:**

(C_25_H_22_P)[Sn(C_6_H_5_)_3_Cl_2_]	*F*(000) = 1576
*M**_r_* = 774.29	*D*_x_ = 1.406 Mg m^−^^3^
Monoclinic, *P*2_1_/*c*	Mo *K*α radiation, λ = 0.71073 Å
Hall symbol: -P 2ybc	Cell parameters from 57161 reflections
*a* = 10.0222 (2) Å	θ = 1.0–28.7°
*b* = 17.1480 (3) Å	µ = 0.92 mm^−^^1^
*c* = 21.2925 (4) Å	*T* = 293 K
β = 92.042 (1)°	Prism, colorless
*V* = 3657.02 (12) Å^3^	0.25 × 0.25 × 0.25 mm
*Z* = 4	

### Data collection

**Table:**

Nonius KappaCCD diffractometer	9370 independent reflections
Radiation source: fine-focus sealed tube	6243 reflections with *I* > 2σ(*I*)
Graphite monochromator	*R*_int_ = 0.072
φ scans, andω scans with κ offset	θ_max_ = 28.6°, θ_min_ = 1.9°
Absorption correction: multi-scan (*SCALEPACK*; Otwinowski & Minor, 1997)	*h* = −13→13
*T*_min_ = 0.803, *T*_max_ = 0.803	*k* = −23→23
29025 measured reflections	*l* = −28→24

### Refinement

**Table:**

Refinement on *F*^2^	Secondary atom site location: difference Fourier map
Least-squares matrix: full	Hydrogen site location: inferred from neighbouring sites
*R*[*F*^2^ > 2σ(*F*^2^)] = 0.039	H-atom parameters constrained
*wR*(*F*^2^) = 0.102	*w* = 1/[σ^2^(*F*_o_^2^) + (0.0443*P*)^2^] where *P* = (*F*_o_^2^ + 2*F*_c_^2^)/3
*S* = 1.03	(Δ/σ)_max_ = 0.003
9370 reflections	Δρ_max_ = 0.80 e Å^−^^3^
425 parameters	Δρ_min_ = −0.76 e Å^−^^3^
0 restraints	Extinction correction: *SHELXS97* (Sheldrick, 2008), Fc^*^=kFc[1+0.001xFc^2^λ^3^/sin(2θ)]^-1/4^
Primary atom site location: structure-invariant direct methods	Extinction coefficient: 0.00069 (19)

### Special details

**Table:**

Geometry. All s.u.'s (except the s.u. in the dihedral angle between two l.s. planes) are estimated using the full covariance matrix. The cell s.u.'s are taken into account individually in the estimation of s.u.'s in distances, angles and torsion angles; correlations between s.u.'s in cell parameters are only used when they are defined by crystal symmetry. An approximate (isotropic) treatment of cell s.u.'s is used for estimating s.u.'s involving l.s. planes.
Refinement. Refinement of *F*^2^ against ALL reflections. The weighted *R*-factor *wR* and goodness of fit *S* are based on *F*^2^, conventional *R*-factors *R* are based on *F*, with *F* set to zero for negative *F*^2^. The threshold expression of *F*^2^ > 2σ(*F*^2^) is used only for calculating *R*-factors(gt) *etc*. and is not relevant to the choice of reflections for refinement. *R*-factors based on *F*^2^ are statistically about twice as large as those based on *F*, and *R*- factors based on ALL data will be even larger.

### Fractional atomic coordinates and isotropic or equivalent isotropic displacement parameters (Å^2^)

**Table:**

	*x*	*y*	*z*	*U*_iso_*/*U*_eq_	
Sn1	0.056967 (18)	0.189828 (11)	0.831152 (8)	0.03831 (8)	
P1	0.60102 (8)	0.16081 (5)	0.49141 (3)	0.04170 (18)	
Cl2	0.12400 (8)	0.07857 (4)	0.75709 (4)	0.05285 (19)	
Cl1	−0.00860 (8)	0.29530 (5)	0.91305 (4)	0.05139 (19)	
C1	0.1426 (3)	0.27746 (17)	0.77245 (13)	0.0422 (6)	
C2	0.0886 (3)	0.35192 (18)	0.76919 (15)	0.0514 (7)	
H2	0.0196	0.3652	0.7952	0.062*	
C6	0.2473 (3)	0.2593 (2)	0.73420 (15)	0.0547 (8)	
H6	0.2851	0.2098	0.7356	0.066*	
C5	0.2958 (4)	0.3156 (2)	0.69355 (17)	0.0676 (10)	
H5	0.3669	0.3036	0.6683	0.081*	
C4	0.2394 (4)	0.3881 (2)	0.69064 (17)	0.0684 (10)	
H4	0.2717	0.4251	0.6631	0.082*	
C3	0.1358 (4)	0.4067 (2)	0.72789 (16)	0.0619 (9)	
H3	0.0972	0.4559	0.7254	0.074*	
C38	0.7056 (3)	0.30371 (17)	0.53593 (14)	0.0496 (7)	
C37	0.6618 (3)	0.25886 (18)	0.47796 (14)	0.0507 (7)	
H37A	0.7366	0.2557	0.4504	0.061*	
H37B	0.5917	0.2882	0.4560	0.061*	
C43	0.8381 (4)	0.3060 (2)	0.55450 (17)	0.0669 (10)	
H43	0.8998	0.2784	0.5316	0.080*	
C7	−0.1504 (3)	0.16911 (17)	0.81155 (14)	0.0432 (6)	
C12	−0.2192 (3)	0.1080 (2)	0.83806 (17)	0.0599 (9)	
H12	−0.1744	0.0741	0.8656	0.072*	
C8	−0.2210 (4)	0.2173 (2)	0.77078 (19)	0.0694 (10)	
H8	−0.1775	0.2590	0.7525	0.083*	
C10	−0.4208 (4)	0.1446 (2)	0.7825 (2)	0.0746 (11)	
H10	−0.5105	0.1357	0.7722	0.090*	
C11	−0.3545 (4)	0.0967 (2)	0.8240 (2)	0.0748 (11)	
H11	−0.3998	0.0562	0.8430	0.090*	
C13	0.1596 (3)	0.12843 (16)	0.90599 (13)	0.0434 (6)	
C14	0.0930 (4)	0.1035 (2)	0.95780 (15)	0.0612 (9)	
H14	0.0025	0.1146	0.9606	0.073*	
C18	0.2933 (3)	0.1121 (2)	0.90324 (16)	0.0621 (9)	
H18	0.3402	0.1276	0.8685	0.074*	
C39	0.6143 (4)	0.3431 (2)	0.57196 (17)	0.0664 (9)	
H39	0.5235	0.3409	0.5614	0.080*	
C42	0.8818 (4)	0.3480 (3)	0.60595 (19)	0.0879 (14)	
H42	0.9721	0.3490	0.6175	0.106*	
C17	0.3594 (4)	0.0720 (3)	0.9525 (2)	0.0814 (12)	
H17	0.4506	0.0623	0.9510	0.098*	
C15	0.1576 (4)	0.0626 (2)	1.00562 (18)	0.0780 (11)	
H15	0.1102	0.0458	1.0398	0.094*	
C16	0.2897 (5)	0.0469 (2)	1.00310 (19)	0.0824 (12)	
H16	0.3330	0.0193	1.0354	0.099*	
C9	−0.3557 (4)	0.2053 (3)	0.7564 (2)	0.0876 (13)	
H9	−0.4013	0.2388	0.7288	0.105*	
C40	0.6596 (5)	0.3860 (2)	0.62403 (19)	0.0838 (13)	
H40	0.5990	0.4131	0.6479	0.101*	
C41	0.7921 (5)	0.3884 (3)	0.64018 (19)	0.0862 (13)	
H41	0.8217	0.4177	0.6747	0.103*	
C31	0.4480 (3)	0.16491 (17)	0.53283 (13)	0.0420 (6)	
C32	0.3462 (3)	0.2142 (2)	0.51172 (16)	0.0571 (8)	
H32	0.3594	0.2474	0.4779	0.068*	
C36	0.4276 (3)	0.11637 (18)	0.58369 (14)	0.0507 (7)	
H36	0.4951	0.0832	0.5984	0.061*	
C34	0.2063 (4)	0.1663 (2)	0.59126 (18)	0.0657 (10)	
H34	0.1251	0.1670	0.6111	0.079*	
C33	0.2255 (3)	0.2142 (2)	0.54078 (17)	0.0659 (9)	
H33	0.1569	0.2467	0.5261	0.079*	
C35	0.3058 (4)	0.1177 (2)	0.61241 (16)	0.0620 (9)	
H35	0.2917	0.0852	0.6465	0.074*	
C25	0.5706 (3)	0.11860 (18)	0.41469 (13)	0.0460 (7)	
C26	0.4456 (3)	0.0926 (2)	0.39523 (15)	0.0566 (8)	
H26	0.3747	0.0959	0.4221	0.068*	
C30	0.6763 (3)	0.1134 (2)	0.37403 (14)	0.0582 (8)	
H30	0.7605	0.1318	0.3863	0.070*	
C27	0.4260 (4)	0.0616 (2)	0.33573 (17)	0.0699 (10)	
H27	0.3416	0.0447	0.3223	0.084*	
C28	0.5301 (4)	0.0557 (2)	0.29691 (16)	0.0731 (11)	
H28	0.5161	0.0343	0.2571	0.088*	
C29	0.6545 (4)	0.0806 (2)	0.31523 (16)	0.0710 (10)	
H29	0.7249	0.0755	0.2882	0.085*	
C19	0.7220 (3)	0.10487 (17)	0.53669 (14)	0.0438 (6)	
C20	0.7749 (3)	0.03695 (19)	0.51251 (16)	0.0532 (8)	
H20	0.7490	0.0203	0.4723	0.064*	
C24	0.7603 (3)	0.1281 (2)	0.59706 (15)	0.0580 (8)	
H24	0.7233	0.1726	0.6142	0.070*	
C21	0.8664 (3)	−0.0061 (2)	0.54841 (19)	0.0671 (10)	
H21	0.9012	−0.0521	0.5325	0.080*	
C23	0.8526 (4)	0.0853 (2)	0.63147 (17)	0.0710 (10)	
H23	0.8792	0.1018	0.6716	0.085*	
C22	0.9059 (4)	0.0190 (3)	0.6076 (2)	0.0727 (11)	
H22	0.9689	−0.0092	0.6312	0.087*	

### Atomic displacement parameters (Å^2^)

**Table:**

	*U*^11^	*U*^22^	*U*^33^	*U*^12^	*U*^13^	*U*^23^
Sn1	0.03712 (11)	0.04086 (12)	0.03680 (11)	0.00303 (8)	−0.00100 (7)	0.00082 (8)
P1	0.0413 (4)	0.0461 (4)	0.0374 (4)	−0.0044 (3)	−0.0027 (3)	0.0008 (3)
Cl2	0.0652 (5)	0.0463 (4)	0.0473 (4)	0.0065 (4)	0.0060 (3)	−0.0056 (3)
Cl1	0.0534 (4)	0.0544 (4)	0.0463 (4)	0.0097 (4)	0.0002 (3)	−0.0090 (3)
C1	0.0402 (15)	0.0449 (15)	0.0413 (15)	−0.0045 (13)	−0.0026 (12)	−0.0017 (13)
C2	0.0552 (18)	0.0433 (17)	0.0557 (18)	0.0043 (15)	0.0008 (14)	−0.0006 (14)
C6	0.0542 (19)	0.0526 (19)	0.0577 (19)	−0.0007 (16)	0.0095 (15)	0.0025 (15)
C5	0.066 (2)	0.079 (3)	0.059 (2)	−0.021 (2)	0.0152 (18)	−0.0001 (18)
C4	0.089 (3)	0.058 (2)	0.058 (2)	−0.027 (2)	−0.0060 (19)	0.0144 (17)
C3	0.080 (2)	0.0432 (17)	0.061 (2)	−0.0042 (18)	−0.0120 (18)	0.0099 (16)
C38	0.0605 (19)	0.0449 (17)	0.0435 (16)	−0.0105 (15)	0.0022 (14)	−0.0030 (13)
C37	0.0569 (18)	0.0494 (17)	0.0461 (17)	−0.0106 (15)	0.0034 (14)	−0.0005 (14)
C43	0.056 (2)	0.083 (3)	0.062 (2)	−0.0204 (19)	0.0075 (16)	−0.0217 (19)
C7	0.0382 (14)	0.0452 (16)	0.0459 (16)	0.0028 (13)	−0.0020 (12)	−0.0035 (13)
C12	0.0490 (18)	0.0528 (19)	0.077 (2)	−0.0025 (16)	−0.0096 (16)	0.0087 (17)
C8	0.0503 (19)	0.070 (2)	0.086 (3)	−0.0014 (18)	−0.0163 (18)	0.025 (2)
C10	0.0407 (18)	0.081 (3)	0.101 (3)	−0.001 (2)	−0.0126 (19)	−0.012 (2)
C11	0.048 (2)	0.067 (2)	0.110 (3)	−0.0116 (19)	0.001 (2)	0.004 (2)
C13	0.0453 (16)	0.0420 (15)	0.0424 (15)	0.0074 (13)	−0.0072 (12)	−0.0028 (13)
C14	0.062 (2)	0.070 (2)	0.0514 (19)	0.0067 (18)	−0.0014 (15)	0.0151 (17)
C18	0.0497 (18)	0.077 (2)	0.058 (2)	0.0162 (18)	−0.0059 (15)	−0.0075 (18)
C39	0.070 (2)	0.059 (2)	0.070 (2)	0.0042 (19)	0.0032 (19)	−0.0080 (18)
C42	0.077 (3)	0.115 (4)	0.071 (3)	−0.035 (3)	−0.002 (2)	−0.025 (3)
C17	0.068 (2)	0.095 (3)	0.079 (3)	0.036 (2)	−0.025 (2)	−0.011 (2)
C15	0.096 (3)	0.082 (3)	0.056 (2)	0.003 (2)	−0.008 (2)	0.021 (2)
C16	0.106 (3)	0.077 (3)	0.062 (2)	0.029 (3)	−0.030 (2)	0.007 (2)
C9	0.054 (2)	0.094 (3)	0.113 (4)	0.012 (2)	−0.028 (2)	0.026 (3)
C40	0.117 (4)	0.073 (3)	0.063 (2)	0.007 (3)	0.018 (2)	−0.019 (2)
C41	0.116 (4)	0.084 (3)	0.058 (2)	−0.032 (3)	−0.003 (2)	−0.023 (2)
C31	0.0397 (15)	0.0454 (15)	0.0405 (15)	−0.0030 (13)	−0.0018 (12)	0.0029 (12)
C32	0.0489 (18)	0.067 (2)	0.0549 (19)	0.0024 (16)	−0.0030 (14)	0.0130 (17)
C36	0.0597 (19)	0.0452 (17)	0.0476 (17)	0.0024 (15)	0.0076 (14)	0.0035 (13)
C34	0.051 (2)	0.076 (2)	0.071 (2)	−0.0071 (19)	0.0140 (17)	−0.015 (2)
C33	0.0440 (18)	0.083 (3)	0.070 (2)	0.0124 (18)	−0.0071 (16)	−0.007 (2)
C35	0.074 (2)	0.056 (2)	0.057 (2)	−0.0088 (19)	0.0228 (18)	−0.0001 (16)
C25	0.0502 (16)	0.0496 (17)	0.0378 (15)	−0.0092 (14)	−0.0041 (12)	−0.0003 (13)
C26	0.0509 (18)	0.068 (2)	0.0503 (18)	−0.0128 (17)	−0.0074 (14)	0.0029 (16)
C30	0.0579 (19)	0.068 (2)	0.0485 (18)	−0.0169 (18)	−0.0013 (15)	−0.0095 (16)
C27	0.077 (2)	0.076 (2)	0.055 (2)	−0.030 (2)	−0.0251 (18)	0.0039 (18)
C28	0.107 (3)	0.070 (2)	0.0406 (18)	−0.024 (2)	−0.0170 (19)	−0.0041 (17)
C29	0.091 (3)	0.080 (3)	0.0430 (18)	−0.018 (2)	0.0107 (18)	−0.0062 (18)
C19	0.0385 (14)	0.0457 (16)	0.0468 (16)	−0.0023 (13)	−0.0028 (12)	0.0033 (13)
C20	0.0506 (18)	0.0546 (19)	0.0543 (18)	−0.0031 (16)	0.0009 (14)	−0.0032 (15)
C24	0.063 (2)	0.059 (2)	0.0512 (18)	0.0004 (17)	−0.0140 (15)	−0.0041 (16)
C21	0.055 (2)	0.056 (2)	0.091 (3)	0.0112 (18)	0.0097 (19)	0.014 (2)
C23	0.069 (2)	0.083 (3)	0.058 (2)	−0.009 (2)	−0.0234 (18)	0.013 (2)
C22	0.054 (2)	0.081 (3)	0.082 (3)	0.005 (2)	−0.0111 (19)	0.026 (2)

### Geometric parameters (Å, º)

**Table:**

Sn1—C7	2.135 (3)	C42—C41	1.366 (6)
Sn1—C13	2.142 (3)	C42—H42	0.9300
Sn1—C1	2.153 (3)	C17—C16	1.373 (6)
Sn1—Cl2	2.5795 (7)	C17—H17	0.9300
Sn1—Cl1	2.6127 (7)	C15—C16	1.354 (6)
P1—C31	1.797 (3)	C15—H15	0.9300
P1—C19	1.799 (3)	C16—H16	0.9300
P1—C25	1.803 (3)	C9—H9	0.9300
P1—C37	1.814 (3)	C40—C41	1.360 (6)
C1—C6	1.386 (4)	C40—H40	0.9300
C1—C2	1.388 (4)	C41—H41	0.9300
C2—C3	1.381 (4)	C31—C36	1.387 (4)
C2—H2	0.9300	C31—C32	1.388 (4)
C6—C5	1.395 (5)	C32—C33	1.378 (4)
C6—H6	0.9300	C32—H32	0.9300
C5—C4	1.368 (5)	C36—C35	1.384 (4)
C5—H5	0.9300	C36—H36	0.9300
C4—C3	1.366 (5)	C34—C35	1.364 (5)
C4—H4	0.9300	C34—C33	1.372 (5)
C3—H3	0.9300	C34—H34	0.9300
C38—C43	1.373 (5)	C33—H33	0.9300
C38—C39	1.390 (5)	C35—H35	0.9300
C38—C37	1.506 (4)	C25—C26	1.380 (4)
C37—H37A	0.9700	C25—C30	1.395 (4)
C37—H37B	0.9700	C26—C27	1.381 (5)
C43—C42	1.370 (5)	C26—H26	0.9300
C43—H43	0.9300	C30—C29	1.383 (5)
C7—C8	1.377 (4)	C30—H30	0.9300
C7—C12	1.386 (4)	C27—C28	1.357 (5)
C12—C11	1.392 (5)	C27—H27	0.9300
C12—H12	0.9300	C28—C29	1.362 (5)
C8—C9	1.389 (5)	C28—H28	0.9300
C8—H8	0.9300	C29—H29	0.9300
C10—C9	1.359 (6)	C19—C20	1.386 (4)
C10—C11	1.361 (6)	C19—C24	1.387 (4)
C10—H10	0.9300	C20—C21	1.386 (5)
C11—H11	0.9300	C20—H20	0.9300
C13—C18	1.372 (4)	C24—C23	1.372 (5)
C13—C14	1.378 (4)	C24—H24	0.9300
C14—C15	1.379 (5)	C21—C22	1.376 (5)
C14—H14	0.9300	C21—H21	0.9300
C18—C17	1.401 (5)	C23—C22	1.363 (6)
C18—H18	0.9300	C23—H23	0.9300
C39—C40	1.393 (5)	C22—H22	0.9300
C39—H39	0.9300		
			
C7—Sn1—C13	120.13 (11)	C41—C42—H42	120.1
C7—Sn1—C1	114.09 (11)	C43—C42—H42	120.1
C13—Sn1—C1	125.74 (11)	C16—C17—C18	120.0 (4)
C7—Sn1—Cl2	91.73 (8)	C16—C17—H17	120.0
C13—Sn1—Cl2	87.85 (8)	C18—C17—H17	120.0
C1—Sn1—Cl2	92.49 (8)	C16—C15—C14	120.4 (4)
C7—Sn1—Cl1	88.83 (8)	C16—C15—H15	119.8
C13—Sn1—Cl1	88.24 (8)	C14—C15—H15	119.8
C1—Sn1—Cl1	91.11 (8)	C15—C16—C17	119.6 (4)
Cl2—Sn1—Cl1	175.77 (3)	C15—C16—H16	120.2
C31—P1—C19	109.17 (13)	C17—C16—H16	120.2
C31—P1—C25	109.91 (14)	C10—C9—C8	120.0 (4)
C19—P1—C25	111.14 (14)	C10—C9—H9	120.0
C31—P1—C37	109.76 (14)	C8—C9—H9	120.0
C19—P1—C37	110.82 (15)	C41—C40—C39	120.4 (4)
C25—P1—C37	106.01 (14)	C41—C40—H40	119.8
C6—C1—C2	118.6 (3)	C39—C40—H40	119.8
C6—C1—Sn1	120.7 (2)	C40—C41—C42	120.3 (4)
C2—C1—Sn1	120.6 (2)	C40—C41—H41	119.9
C3—C2—C1	121.0 (3)	C42—C41—H41	119.9
C3—C2—H2	119.5	C36—C31—C32	119.4 (3)
C1—C2—H2	119.5	C36—C31—P1	120.8 (2)
C1—C6—C5	119.9 (3)	C32—C31—P1	119.7 (2)
C1—C6—H6	120.1	C33—C32—C31	120.1 (3)
C5—C6—H6	120.1	C33—C32—H32	120.0
C4—C5—C6	120.3 (3)	C31—C32—H32	120.0
C4—C5—H5	119.9	C35—C36—C31	119.5 (3)
C6—C5—H5	119.9	C35—C36—H36	120.3
C3—C4—C5	120.5 (3)	C31—C36—H36	120.3
C3—C4—H4	119.8	C35—C34—C33	120.2 (3)
C5—C4—H4	119.8	C35—C34—H34	119.9
C4—C3—C2	119.7 (3)	C33—C34—H34	119.9
C4—C3—H3	120.1	C34—C33—C32	120.1 (3)
C2—C3—H3	120.1	C34—C33—H33	119.9
C43—C38—C39	118.4 (3)	C32—C33—H33	119.9
C43—C38—C37	120.1 (3)	C34—C35—C36	120.7 (3)
C39—C38—C37	121.5 (3)	C34—C35—H35	119.7
C38—C37—P1	115.7 (2)	C36—C35—H35	119.7
C38—C37—H37A	108.4	C26—C25—C30	119.6 (3)
P1—C37—H37A	108.4	C26—C25—P1	121.6 (2)
C38—C37—H37B	108.4	C30—C25—P1	118.7 (2)
P1—C37—H37B	108.4	C25—C26—C27	119.8 (3)
H37A—C37—H37B	107.4	C25—C26—H26	120.1
C42—C43—C38	121.6 (4)	C27—C26—H26	120.1
C42—C43—H43	119.2	C29—C30—C25	119.3 (3)
C38—C43—H43	119.2	C29—C30—H30	120.3
C8—C7—C12	117.3 (3)	C25—C30—H30	120.3
C8—C7—Sn1	119.9 (2)	C28—C27—C26	120.0 (3)
C12—C7—Sn1	122.8 (2)	C28—C27—H27	120.0
C7—C12—C11	120.8 (3)	C26—C27—H27	120.0
C7—C12—H12	119.6	C27—C28—C29	121.2 (3)
C11—C12—H12	119.6	C27—C28—H28	119.4
C7—C8—C9	121.6 (4)	C29—C28—H28	119.4
C7—C8—H8	119.2	C28—C29—C30	120.0 (3)
C9—C8—H8	119.2	C28—C29—H29	120.0
C9—C10—C11	119.9 (4)	C30—C29—H29	120.0
C9—C10—H10	120.1	C20—C19—C24	119.2 (3)
C11—C10—H10	120.1	C20—C19—P1	120.6 (2)
C10—C11—C12	120.3 (4)	C24—C19—P1	120.2 (2)
C10—C11—H11	119.8	C21—C20—C19	119.8 (3)
C12—C11—H11	119.8	C21—C20—H20	120.1
C18—C13—C14	118.2 (3)	C19—C20—H20	120.1
C18—C13—Sn1	120.8 (2)	C23—C24—C19	120.1 (3)
C14—C13—Sn1	120.9 (2)	C23—C24—H24	120.0
C13—C14—C15	121.4 (3)	C19—C24—H24	120.0
C13—C14—H14	119.3	C22—C21—C20	120.1 (4)
C15—C14—H14	119.3	C22—C21—H21	120.0
C13—C18—C17	120.3 (3)	C20—C21—H21	120.0
C13—C18—H18	119.8	C22—C23—C24	120.8 (4)
C17—C18—H18	119.8	C22—C23—H23	119.6
C38—C39—C40	119.6 (4)	C24—C23—H23	119.6
C38—C39—H39	120.2	C23—C22—C21	120.0 (4)
C40—C39—H39	120.2	C23—C22—H22	120.0
C41—C42—C43	119.7 (4)	C21—C22—H22	120.0
			
C7—Sn1—C1—C6	−119.9 (3)	C13—C18—C17—C16	1.8 (6)
C13—Sn1—C1—C6	62.3 (3)	C13—C14—C15—C16	1.0 (6)
Cl2—Sn1—C1—C6	−26.9 (3)	C14—C15—C16—C17	0.0 (7)
Cl1—Sn1—C1—C6	150.9 (2)	C18—C17—C16—C15	−1.4 (7)
C7—Sn1—C1—C2	56.0 (3)	C11—C10—C9—C8	1.5 (7)
C13—Sn1—C1—C2	−121.8 (2)	C7—C8—C9—C10	−0.2 (7)
Cl2—Sn1—C1—C2	149.0 (2)	C38—C39—C40—C41	−1.0 (6)
Cl1—Sn1—C1—C2	−33.2 (2)	C39—C40—C41—C42	−0.9 (7)
C6—C1—C2—C3	1.4 (5)	C43—C42—C41—C40	1.1 (7)
Sn1—C1—C2—C3	−174.7 (3)	C19—P1—C31—C36	−12.8 (3)
C2—C1—C6—C5	−0.1 (5)	C25—P1—C31—C36	109.3 (3)
Sn1—C1—C6—C5	175.9 (3)	C37—P1—C31—C36	−134.4 (3)
C1—C6—C5—C4	−0.9 (6)	C19—P1—C31—C32	170.6 (3)
C6—C5—C4—C3	0.7 (6)	C25—P1—C31—C32	−67.2 (3)
C5—C4—C3—C2	0.6 (6)	C37—P1—C31—C32	49.0 (3)
C1—C2—C3—C4	−1.6 (5)	C36—C31—C32—C33	−0.7 (5)
C43—C38—C37—P1	96.7 (3)	P1—C31—C32—C33	175.9 (3)
C39—C38—C37—P1	−83.0 (4)	C32—C31—C36—C35	0.2 (5)
C31—P1—C37—C38	66.4 (3)	P1—C31—C36—C35	−176.3 (3)
C19—P1—C37—C38	−54.3 (3)	C35—C34—C33—C32	−0.9 (6)
C25—P1—C37—C38	−174.9 (2)	C31—C32—C33—C34	1.0 (6)
C39—C38—C43—C42	−2.2 (6)	C33—C34—C35—C36	0.5 (6)
C37—C38—C43—C42	178.2 (4)	C31—C36—C35—C34	−0.1 (5)
C13—Sn1—C7—C8	169.3 (3)	C31—P1—C25—C26	−1.8 (3)
C1—Sn1—C7—C8	−8.7 (3)	C19—P1—C25—C26	119.1 (3)
Cl2—Sn1—C7—C8	−102.2 (3)	C37—P1—C25—C26	−120.4 (3)
Cl1—Sn1—C7—C8	82.0 (3)	C31—P1—C25—C30	177.3 (3)
C13—Sn1—C7—C12	−11.1 (3)	C19—P1—C25—C30	−61.8 (3)
C1—Sn1—C7—C12	171.0 (2)	C37—P1—C25—C30	58.7 (3)
Cl2—Sn1—C7—C12	77.5 (3)	C30—C25—C26—C27	0.0 (5)
Cl1—Sn1—C7—C12	−98.4 (3)	P1—C25—C26—C27	179.1 (3)
C8—C7—C12—C11	−0.4 (5)	C26—C25—C30—C29	−1.4 (5)
Sn1—C7—C12—C11	180.0 (3)	P1—C25—C30—C29	179.5 (3)
C12—C7—C8—C9	−0.3 (6)	C25—C26—C27—C28	1.0 (6)
Sn1—C7—C8—C9	179.3 (3)	C26—C27—C28—C29	−0.6 (6)
C9—C10—C11—C12	−2.2 (7)	C27—C28—C29—C30	−0.8 (6)
C7—C12—C11—C10	1.7 (6)	C25—C30—C29—C28	1.8 (6)
C7—Sn1—C13—C18	150.4 (2)	C31—P1—C19—C20	118.8 (2)
C1—Sn1—C13—C18	−31.9 (3)	C25—P1—C19—C20	−2.5 (3)
Cl2—Sn1—C13—C18	59.6 (3)	C37—P1—C19—C20	−120.1 (2)
Cl1—Sn1—C13—C18	−122.0 (3)	C31—P1—C19—C24	−59.2 (3)
C7—Sn1—C13—C14	−28.9 (3)	C25—P1—C19—C24	179.4 (2)
C1—Sn1—C13—C14	148.8 (2)	C37—P1—C19—C24	61.8 (3)
Cl2—Sn1—C13—C14	−119.6 (3)	C24—C19—C20—C21	−1.0 (5)
Cl1—Sn1—C13—C14	58.7 (3)	P1—C19—C20—C21	−179.0 (2)
C18—C13—C14—C15	−0.6 (5)	C20—C19—C24—C23	2.0 (5)
Sn1—C13—C14—C15	178.7 (3)	P1—C19—C24—C23	−179.9 (3)
C14—C13—C18—C17	−0.9 (5)	C19—C20—C21—C22	−0.8 (5)
Sn1—C13—C18—C17	179.9 (3)	C19—C24—C23—C22	−1.3 (6)
C43—C38—C39—C40	2.4 (5)	C24—C23—C22—C21	−0.5 (6)
C37—C38—C39—C40	−177.9 (3)	C20—C21—C22—C23	1.5 (6)
C38—C43—C42—C41	0.4 (7)		

### Hydrogen-bond geometry (Å, º)

**Table:**

*D*—H···*A*	*D*—H	H···*A*	*D*···*A*	*D*—H···*A*
C35—H35···Cl2	0.93	2.94	3.696 (3)	139
C37—H37*A*···Cl1^i^	0.97	2.84	3.743 (3)	155
C30—H30···Cl1^i^	0.93	2.67	3.596 (3)	171

Symmetry code: (i) *x*+1, −*y*+1/2, *z*−1/2.
